# Supplementary material for: Modeling glioblastoma heterogeneity as a dynamic network of cell states
Source: Mol Syst Biol. 2021 Sep 16;17(9):e10105. doi: 10.15252/msb.202010105 (PMC8444284; doi:10.15252/msb.202010105)
Supplement: Supplementary file 6 — Source Data for Figure 5 [file MSB-17-e10105-s004.zip › Figure5A_sourcedata/GSEA_3017/hallmarks_stateF.GseaPreranked.1621934628066/gsea_report_for_na_pos_1621934628066.html]

Report for na\_pos 1621934628066 [GSEA]

| GS  follow link to MSigDB | GS DETAILS | SIZE | ES | NES | NOM p-val | FDR q-val | FWER p-val | RANK AT MAX | LEADING EDGE || 1 | HALLMARK\_P53\_PATHWAY | Details ... | 69 | 0.54 | 3.11 | 0.000 | 0.000 | 0.000 | 473 | tags=58%, list=21%, signal=71% |
| 2 | HALLMARK\_TNFA\_SIGNALING\_VIA\_NFKB | Details ... | 52 | 0.50 | 2.63 | 0.000 | 0.000 | 0.000 | 624 | tags=62%, list=28%, signal=83% |
| 3 | HALLMARK\_INFLAMMATORY\_RESPONSE | Details ... | 27 | 0.57 | 2.48 | 0.000 | 0.000 | 0.000 | 210 | tags=41%, list=9%, signal=44% |
| 4 | HALLMARK\_MYOGENESIS | Details ... | 44 | 0.48 | 2.42 | 0.000 | 0.000 | 0.000 | 467 | tags=48%, list=21%, signal=59% |
| 5 | HALLMARK\_COMPLEMENT | Details ... | 33 | 0.47 | 2.26 | 0.000 | 0.001 | 0.004 | 426 | tags=48%, list=19%, signal=59% |
| 6 | HALLMARK\_HYPOXIA | Details ... | 48 | 0.42 | 2.20 | 0.000 | 0.002 | 0.010 | 628 | tags=56%, list=28%, signal=76% |
| 7 | HALLMARK\_INTERFERON\_GAMMA\_RESPONSE | Details ... | 36 | 0.47 | 2.18 | 0.000 | 0.002 | 0.011 | 267 | tags=33%, list=12%, signal=37% |
| 8 | HALLMARK\_ADIPOGENESIS | Details ... | 33 | 0.46 | 2.15 | 0.004 | 0.002 | 0.013 | 431 | tags=42%, list=19%, signal=52% |
| 9 | HALLMARK\_APOPTOSIS | Details ... | 41 | 0.43 | 2.14 | 0.000 | 0.002 | 0.015 | 569 | tags=59%, list=25%, signal=77% |
| 10 | HALLMARK\_IL2\_STAT5\_SIGNALING | Details ... | 38 | 0.44 | 2.11 | 0.000 | 0.002 | 0.016 | 470 | tags=47%, list=21%, signal=59% |
| 11 | HALLMARK\_XENOBIOTIC\_METABOLISM | Details ... | 37 | 0.43 | 2.08 | 0.000 | 0.003 | 0.026 | 767 | tags=68%, list=34%, signal=101% |
| 12 | HALLMARK\_COAGULATION | Details ... | 26 | 0.47 | 2.00 | 0.000 | 0.006 | 0.053 | 357 | tags=46%, list=16%, signal=54% |
| 13 | HALLMARK\_ESTROGEN\_RESPONSE\_EARLY | Details ... | 30 | 0.44 | 1.98 | 0.002 | 0.007 | 0.064 | 592 | tags=60%, list=26%, signal=80% |
| 14 | HALLMARK\_KRAS\_SIGNALING\_UP | Details ... | 27 | 0.44 | 1.94 | 0.002 | 0.008 | 0.083 | 745 | tags=63%, list=33%, signal=93% |
| 15 | HALLMARK\_INTERFERON\_ALPHA\_RESPONSE | Details ... | 20 | 0.45 | 1.84 | 0.007 | 0.017 | 0.168 | 624 | tags=55%, list=28%, signal=75% |
| 16 | HALLMARK\_IL6\_JAK\_STAT3\_SIGNALING | Details ... | 17 | 0.48 | 1.81 | 0.006 | 0.018 | 0.190 | 333 | tags=47%, list=15%, signal=55% |
| 17 | HALLMARK\_HEME\_METABOLISM | Details ... | 34 | 0.37 | 1.70 | 0.028 | 0.033 | 0.330 | 583 | tags=50%, list=26%, signal=66% |
| 18 | HALLMARK\_CHOLESTEROL\_HOMEOSTASIS | Details ... | 21 | 0.41 | 1.63 | 0.035 | 0.048 | 0.465 | 271 | tags=29%, list=12%, signal=32% |
| 19 | HALLMARK\_EPITHELIAL\_MESENCHYMAL\_TRANSITION | Details ... | 57 | 0.29 | 1.56 | 0.039 | 0.070 | 0.632 | 366 | tags=32%, list=16%, signal=37% |
| 20 | HALLMARK\_UV\_RESPONSE\_DN | Details ... | 33 | 0.33 | 1.52 | 0.051 | 0.079 | 0.694 | 793 | tags=58%, list=35%, signal=88% |
| 21 | HALLMARK\_PI3K\_AKT\_MTOR\_SIGNALING |  | 20 | 0.36 | 1.41 | 0.104 | 0.135 | 0.882 | 447 | tags=35%, list=20%, signal=43% |
| 22 | HALLMARK\_MTORC1\_SIGNALING |  | 62 | 0.24 | 1.36 | 0.117 | 0.160 | 0.925 | 377 | tags=31%, list=17%, signal=36% |
| 23 | HALLMARK\_PROTEIN\_SECRETION |  | 18 | 0.35 | 1.32 | 0.178 | 0.183 | 0.958 | 937 | tags=78%, list=42%, signal=132% |
| 24 | HALLMARK\_ESTROGEN\_RESPONSE\_LATE |  | 45 | 0.25 | 1.27 | 0.145 | 0.212 | 0.976 | 366 | tags=31%, list=16%, signal=36% |
| 25 | HALLMARK\_ANDROGEN\_RESPONSE |  | 27 | 0.27 | 1.18 | 0.260 | 0.301 | 0.997 | 344 | tags=33%, list=15%, signal=39% |
| 26 | HALLMARK\_FATTY\_ACID\_METABOLISM |  | 28 | 0.26 | 1.17 | 0.266 | 0.296 | 0.997 | 456 | tags=32%, list=20%, signal=40% |
| 27 | HALLMARK\_ALLOGRAFT\_REJECTION |  | 19 | 0.28 | 1.07 | 0.376 | 0.409 | 1.000 | 404 | tags=32%, list=18%, signal=38% |
| 28 | HALLMARK\_OXIDATIVE\_PHOSPHORYLATION |  | 41 | 0.20 | 1.00 | 0.490 | 0.496 | 1.000 | 1488 | tags=85%, list=66%, signal=247% |
| 29 | HALLMARK\_UNFOLDED\_PROTEIN\_RESPONSE |  | 31 | 0.21 | 0.97 | 0.486 | 0.521 | 1.000 | 380 | tags=26%, list=17%, signal=31% |
| 30 | HALLMARK\_APICAL\_JUNCTION |  | 26 | 0.18 | 0.83 | 0.677 | 0.701 | 1.000 | 469 | tags=27%, list=21%, signal=34% |
Table: Gene sets enriched in phenotype **na **[plain text format]****
